# Pneumococcal vaccination: what have we learnt so far and what can we expect in the future?

**DOI:** 10.1007/s10096-014-2208-6

**Published:** 2014-08-23

**Authors:** A. Torres, P. Bonanni, W. Hryniewicz, M. Moutschen, R. R. Reinert, T. Welte

**Affiliations:** 1 Servei de Pneumologia, Hospital Clínic de Barcelona, Institut d’Investigacions Biomèdiques August Pi i Sunyer (IDIBAPS), CIBER de Enfermedades Respiratorias (CIBERes), University of Barcelona, Barcelona, India; 2 Department of Health Sciences, University of Florence, Florence, Italy; 3 National Medicines Institute, Warsaw, Poland; 4 Department of Infectious Diseases and General Internal Medicine, CHU de Liège/University of Liège, Liège, Belgium; 5 Pfizer Vaccines (Medical Development Group and Scientific Affairs), Paris, France; 6 Klinic für Pneumologie, Medizinische Hochschule Hannover, Hannover, Germany

## Abstract

Individuals <2 years and ≥50 years of age, as well as those with other specific risk factors, are especially vulnerable to invasive pneumococcal disease (IPD). Conjugate vaccines have been developed against encapsulated bacteria such as *Streptococcus pneumoniae* to provide improved immune responses. The 7-valent pneumococcal conjugate vaccine (PCV7) has significantly reduced the burden of vaccine-type pneumococcal diseases in children, including invasive disease and pneumonia and acute otitis media. There have also been significant declines in antimicrobial resistance in 7-valent vaccine serotypes and carriage of *S. pneumoniae* in the post-PCV7 era. Two to three years after the introduction of PCV13, there is emerging, global evidence of a reduced burden of pneumococcal diseases in children, including declines in IPD (UK and Germany) and nasopharyngeal carriage of PCV13 serotypes (Portugal and France). The functional immunogenicity of PCV13 in individuals ≥50 years of age has been demonstrated in clinical trials in comparison with the 23-valent pneumococcal polysaccharide vaccine and for children and adults 6 to 49 years of age. Between 2011 and 2013, PCV13 received market authorisation by the European Medicines Agency (EMA) for these additional age groups and is now available in Europe for the prevention of pneumococcal disease in all age groups.

## Introduction: the burden of pneumococcal disease

The aim of this article is to discuss what could be expected from 13-valent pneumococcal conjugate vaccine (PCV13) use in the population over 5 years of age based current knowledge from the post-marketing experience of childhood vaccination with PCV13 and from pivotal trials performed in children, adolescents and adults. We will also review the characteristics of the immune response to conjugate vaccines.

The burden of invasive pneumococcal disease (IPD) is high, especially in individuals <2 years and ≥65 years of age who have the highest incidence and case fatality rate (CFR) [1]. The overall incidence of IPD in Europe in 2010 was 5.2 cases per 100,000 population, with the most affected age groups being <1 year and ≥65 years old (18.5 and 15.6 cases per 100,000, respectively) [2]. In the USA, CFRs for IPD in adults have not changed significantly over the last several decades [3]. Mortality rates due to IPD have remained high despite the availability of clear guidelines for treatment, with a number of highly effective antibiotics [4].

The clinical presentation of IPD varies according to age. Bacteraemia without source or focus of infection (i.e. non-meningitis, non-pneumonia IPD) is the most common presentation in children aged 0–5 years, with an estimated global incidence of 87 cases per 100,000 versus 17 cases per 100,000 for pneumococcal meningitis [5]. In the Netherlands, pneumococcal pneumonia was the most frequent presentation in adults during 2004–2006 [6]. Similar findings were observed in a Spanish hospital-based surveillance study (the ODIN study), in which pneumonia accounted for 70 % of IPD cases in adults older than 18 years [7]. Within this study, CFRs were also found to vary according to clinical presentation, with sepsis being associated with the highest rate (40 %), followed by peritonitis (20 %) and complicated pneumonia (18 %).

Certain comorbidities, including chronic heart, hepatic or pulmonary disease, diabetes mellitus, acquired immunodeficiency syndrome (AIDS) or other immunosuppressions, increase the risk of pneumococcal diseases in both children and adults [8]. A retrospective analysis of 22,000 IPD cases in England (2002–2009) reported a higher risk of IPD-related hospitalisation and mortality in individuals with comorbidities versus those without comorbidities in all age groups. The effect of underlying conditions on the risk of hospitalisation for IPD was highest in children aged 2–15 years, with a nearly 12-fold increase in IPD in those with comorbidities compared with those without (versus nearly 8-fold and 3-fold increases in adults aged 16–64 years and adults aged ≥65 years, respectively). Therefore, despite a significant reduction of cases in populations in the post-PCV era, the risk of IPD is generally still higher in comorbid versus healthy populations and in immunocompromised versus immunocompetent patients. [9–12].

In addition to young and old age and certain comorbidities, environmental, external and behavioural factors may predispose individuals to pneumococcal diseases, as listed in Table 1 [13–15]. Torres et al. suggested that clinical presentations of diseases are different according to the underlying conditions. Pneumonia is common in patients with respiratory diseases and/or in those who smoke, and bacteraemia is common in cancer patients [7].

**Table 1:** Factors associated with an increased risk of pneumococcal diseases

Age	Host factors		External factors	Behavioural
	Immunocompetent	Immunocompromised		
<2 years ≥50 years	Underlying medical conditions • CCVD • CPD • Diabetes • Alcoholism • CLD • Cerebrospinal fluid leaks	HIV CRF, nephrotic syndrome Cancer (solid, haematological) Organ and bone marrow transplant Auto-immune diseases Immunosuppressive therapy, corticosteroids Primary immunodeficiencies Functional and anatomical asplenia	Socioeconomic Environmental • Preceding viral respiratory infection • Residence in an institution (e.g. nursing home) • Frequent contact with children	Smoking Heavy alcohol use

CCVD: cardiovascular and cerebrovascular disease; CPD: chronic pulmonary disease, CLD: chronic liver disease; CRF: chronic renal failure; HIV: human immunodeficiency virus

Pneumococcal pneumonia results in significant morbidity, leading to high rates of hospitalisations, especially in elderly patients. Hospitalisation rates due to pneumococcal pneumonia in Spain were 0.25 per 1,000 in patients aged 50–54 years versus 4.21 per 1,000 in those ≥85 years of age [16]. *Streptococcus pneumoniae* is the leading cause of community-acquired pneumonia (CAP), accounting for about 30 % of cases [17] and, thus, the epidemiology of pneumococcal CAP can be extrapolated from all-cause CAP. Torres et al. reported an increased risk of CAP in men (compared with women), patients ≥65 years of age, patients with certain comorbid conditions, such as previous history of pneumonia, chronic respiratory disease, chronic obstructive pulmonary disease or human immunodeficiency virus (HIV) infection, and patients with specific lifestyle factors, including being underweight, smoking, high alcohol consumption, regular contact with children in day care or poor dental hygiene [18].

## Key milestones in the development of pneumococcal vaccines

Pneumococcal vaccines have been available for more than 100 years, starting with the development of the pneumococcal whole-cell vaccine in 1911 [19], followed by the availability of polysaccharide vaccines with increasing numbers of serotypes, including the 23-valent pneumococcal polysaccharide vaccine (PPV23), which became available in 1983. The 7-valent pneumococcal conjugate vaccine (PCV7), which comprises pneumococcal polysaccharides for serotypes 4, 6B, 9V, 14, 18C, 19F and 23F, was introduced in 2000 in the USA and in 2001 in Europe [20], and has been successfully used in many childhood pneumococcal immunisation programmes around the world. The World Health Organization (WHO) has recently reported on the progress of PCV introductions into national immunisation programmes (NIPs). As of December 2012, 44 % of WHO member states had included PCV in their routine infant immunisation schedule, representing 31 % of all children born in WHO member states [21]. In 2009, higher-valent PCVs became available. In Europe, PCV10 (Synflorix®, comprising the additional serotypes: 1, 5 and 7F) was indicated for active immunisation against IPD and acute otitis media (AOM) in infants and children from 6 weeks up to 5 years of age [22] and in 2013, the pneumonia indication was added. In Europe, PCV13 (Prevenar 13®, comprising the additional serotypes: 1, 3, 5, 6A, 7F and 19A) is indicated for the prevention of IPD, pneumonia and AOM caused by *S. pneumoniae* in infants and children from 6 weeks up to the age of 17 years and for IPD in individuals aged ≥18 years [23]. This reflects the expanded use of PCV13 in individuals ≥6 years of age in Europe. The marketing authorisation for PCV13 for adults aged 50 years or over was received in 2011.

## Conjugate vaccines: what are their attributes?

Conjugate vaccines, comprising a conjugate between an antigenic protein and a polysaccharide, have been developed against a variety of bacterial species, including *S. pneumoniae*, *Neisseria meningitidis* and *Haemophilus influenzae* type b, to overcome the issues associated with the T cell-independent immunological characteristics of pure polysaccharide antigens (reviewed by Blanchard-Rohner and Pollard [24]).

Apart from a few polysaccharides that carry both positive and negative charges, most polysaccharides found in encapsulated bacteria and in polysaccharide vaccines cannot be processed and bound to major histocompatibility complex class II (MHC II) molecules for presentation to T-helper cells and are, therefore, considered T-independent antigens [25, 26]. As a result, B-cell activation is incomplete and generally occurs outside germinal centres. There is limited immunoglobulin (Ig) class switch (mostly IgM and IgG2) [27] and somatic hypermutation (a process that is critical for obtaining high-affinity antibodies) [28]. Most importantly, the memory generated by exposure to such polysaccharides is suboptimal compared with that observed after T-dependent antigen stimulation and is mostly supported by long-lived plasma cells [29] and short-lived memory B cells that differ from T-dependent B cells [30].

B-cell response to pure polysaccharide vaccines is limited to specific subsets, principally B1 cells and marginal zone B cells [31]. B1 cells produce short-lived low-affinity antibody responses that provide the first line of defence against pathogen invasion [24]. Marginal zone B cells are sensitive to ageing and inflammation [32, 33]. Moreover, they are found only in the spleen, which explains why the response to T-independent antigens is limited after splenectomy [34]. Accordingly, increased rates of infections from encapsulated bacteria in patients with asplenia or diminished splenic function have been attributed, in part, to the absence of marginal zone B cells in these patients [24]. Infants <2 years of age are also vulnerable to these infections as, before this age, the marginal zone is immature and unable to support the development of marginal zone B cells [35].

Repeated doses of polysaccharide vaccine administered at intervals of <5 years have been shown to result in subsequently lower antibody levels (a phenomenon known as hyporesponsiveness) due to the depletion of polysaccharide-specific B cells [36, 37]. It has been shown in a study in neonatal mice receiving meningococcal serotype C polysaccharide booster vaccine that this hyporesponsiveness is due to apoptosis of memory B cells [38], possibly by the inhibition of critical survival pathways for B cells [39].

**Fig. 1:**
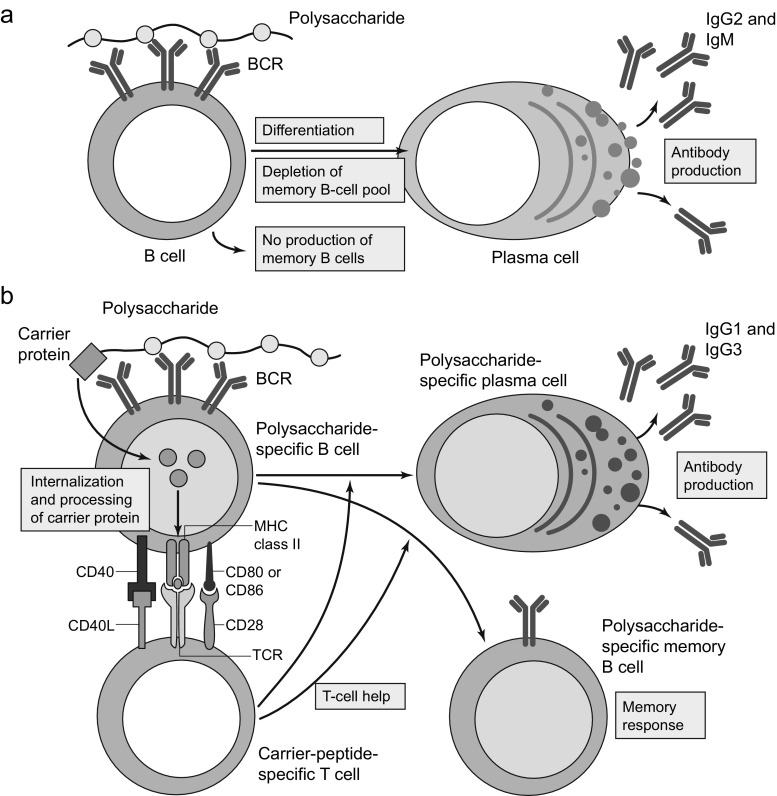
Immune response to polysaccharide and conjugate vaccines. **a** Polysaccharides from the encapsulated bacteria that cause disease in early childhood stimulate B cells by cross‑linking the B‑cell receptor (*BCR*) and drive the production of immunoglobulins. This process results in a lack of production of new memory B cells and a depletion of the memory B‑cell pool, such that subsequent immune responses are decreased. **b** The carrier protein from protein–polysaccharide conjugate vaccines is processed by the polysaccharide‑specific B cell, and peptides are presented to carrier‑peptide‑specific T cells, resulting in T‑cell help for the production of both plasma cells and memory B cells. *CD40L*, CD40 ligand; *TCR*, T‑cell receptor. Reprinted with permission from Macmillan Publishers Ltd.: Pollard et al. Nat Rev Immunol 2009;9(3):213–20 [37], copyright 2009

In contrast with pure polysaccharide vaccines, conjugate vaccines produce an immunological response involving both B cells and T cells (Fig. 1). The chemical conjugation of a carrier protein to the polysaccharide antigen results in activation of the B-cell receptor following polysaccharide binding. The presentation of carrier protein peptides to the T cell in association with MHC II on the B-cell surface also provides signals for the activation of the T-helper cells [24]. This B cell–T cell interaction, which normally takes place during infections, provides the necessary costimulatory signals to B cells to initiate the process of germinal centre reaction [24, 40, 41]. This results in high titres of opsonising antibodies with Ig class switch and somatic hypermutation [24, 28]. New data for conjugate vaccines against *S. agalactiae* (group B *Streptococcus*) suggest that part of the carbohydrate molecule also binds and subsequently activates T cells directed against polysaccharide antigens [42]. In view of the crucial role of T cells in mucosal immunity, this observation could help explain the dramatic effect of conjugate vaccines in the reduction of nasopharyngeal colonisation.

A conjugate vaccine, therefore, is expected to have benefits over a polysaccharide vaccine, in terms of booster response, immunological memory and generally improved immune responses, due to the T cell-dependent characteristics of the immune response.

## PCV immunisation in infants: what have we learnt so far?

PCV7 has significantly reduced the burden of pneumococcal diseases in children. PCV7 was introduced in the USA in 2000. The Centers for Disease Control and Prevention (CDC) reported a 77 % reduction in overall IPD rates and a 98 % reduction in PCV7 serotype disease in children aged <5 years for 2005 compared with pre-PCV7 years (1998–1999), based on an analysis of laboratory and population surveillance data [43]. Reductions in overall and/or PCV7 serotype IPD cases have also been documented in children aged <2 or <5 years in many other countries, including Australia, Canada, France, Norway and Spain, following the introduction of PCV7 [44–49]. Furthermore, reductions in hospitalisation rates for all-cause pneumonia (39 %) and pneumococcal pneumonia (65 %) in children <2 years of age have been observed in an US analysis of admissions data [50]. Similarly, a Polish study found that pneumonia admission rates significantly declined following the introduction of PCV7 in children <5 years of age in the city of Kielce [51]. PCV7 has also reduced otitis media in children aged <2 years, as demonstrated by a ≥28 % reduction in recurrent otitis media [52] and a ≥43 % reduction in AOM outpatient visits or prescriptions [53].

Antibiotic resistance in vaccine serotypes and carriage of *S. pneumoniae* has declined since the introduction of PCV7. Kyaw et al. reported an 81 % decrease in penicillin-resistant IPD (almost all caused by vaccine or vaccine-related serotypes) among children aged <2 years in the USA [54]. In addition, reductions in the carriage of vaccine serotypes and antibiotic-resistant serotypes have been observed in Greece and the USA [55, 56].

PCV7 has demonstrated an indirect effect in unvaccinated populations (herd effect), as exemplified by declines in IPD cases within adults aged ≥65 years and infants ≤60 days of age in Canada and the USA [10, 45]. A review of data from 14 countries, most of which were developed countries, also reported consistent and significant declines in both vaccine-type IPD and vaccine-type pneumococcal carriage following PCV introduction in individuals not targeted for PCV vaccination [57]. These decreases were found to be contemporaneous in studies assessing both vaccine-type carriage and vaccine-type IPD, and longitudinal data demonstrated continued declines, with the greatest declines occurring in the first few years following PCV introduction.

Since the availability of PCV7 in 2000/2001, there have been changes in the overall serotype distribution of *S. pneumoniae*, in particular, a rise in serotype 19A has been observed globally. The estimated proportion of IPD caused by serotype 19A has ranged from 22 % reported in a Spanish study (2001–2005) in 85 vaccinated and unvaccinated individuals <5 years of age to 40 % in a US study (2005) in 1.26 million individuals <5 years of age [43, 58]. A rate of 27 % has also been reported in cases of pneumococcal pneumonia in a French study [59]. This changing serotype epidemiology has led to the development and introduction of higher-valent pneumococcal conjugate vaccines, including PCV13, which includes serotype 19A, to provide improved serotype coverage against pneumococcal diseases.

**Fig. 2:**
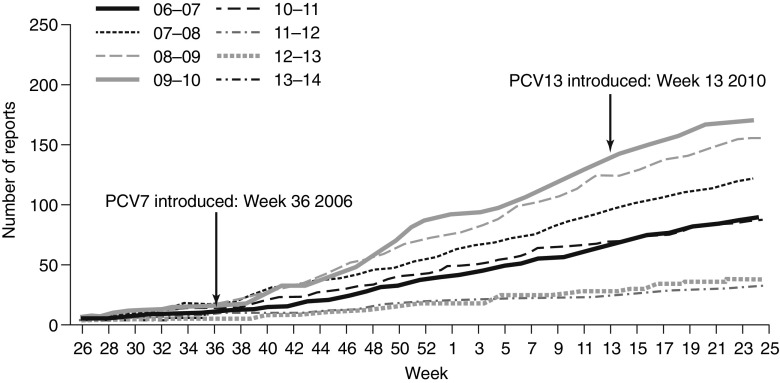
Rates of invasive pneumococcal disease (IPD) caused by six additional serotypes in PCV13* among children aged <2 years (2006–2013). *Serotypes 1, 3, 5, 6A, 7F and 19A. Reprinted with permission from Public Health England [60]. *PCV13*, 13-valent pneumococcal conjugate vaccine

By 2013, 2–3 years after the widespread use of PCV13, there were emerging data concerning the impact of PCV13 on the rate of vaccine serotype-specific IPD in children. There has been a decline in IPD in the UK, due to the six additional serotypes in PCV13 following the introduction of PCV13 in April 2010 (Fig. 2) and a sustained decline in IPD due to shared serotypes in PCV7 and PCV13 [60]. Furthermore, reductions in IPD due to PCV13 serotypes have been reported in children <2 years of age in Germany following the introduction of PCV10 and PCV13 [61]. Temporal trends in nasopharyngeal carriage have also been monitored in several studies following the introduction of PCV13. In a study of 448 healthy children aged ≤6 years attending day-care centres in Portugal, there were reductions of 8 % and 10 %, respectively, in the incidence of nasopharyngeal carriage of serotypes 19A and 6C from 2010 to 2011 following the introduction of PCV13 [62]. Similarly, in a French study involving children <2 years of age with AOM, significant reductions in the nasopharyngeal carriage of PCV13 serotypes were found in PCV13-vaccinated children (*n* = 652) compared with PCV13-unvaccinated individuals (*n* = 290) within 1 year of PCV13 introduction (*p* < 0.001) [63]. This reduction in nasopharyngeal carriage is important for the control of pneumococcal diseases in adults, as children in day care are a key source of transmission to adults, as demonstrated by a US case–control study, which found this to be an independent risk factor for IPD in adults (odds ratio 3.0; 95 % confidence interval [CI] 1.5–6.2; *p* = 0.003) [64].

Similar to the PCV7 experience, in countries where there has been a high uptake of PCV13 within childhood immunisation programmes, there has been a decline in vaccine-type IPD cases in adults, indicating a possible early herd effect. For example, in Norway, which had a vaccine coverage rate of 92 % for children <2 years of age in 2012, IPD cases caused by PCV13 serotypes in individuals ≥65 years old declined from 27/100,000 in 2010 to 18/100,000 in 2012 [65, 66]. There have also been declines in the number of IPD cases caused by the additional PCV13 serotypes (1, 3, 5, 6A, 7F and 19A) in individuals aged ≥5 years in the UK and in adults in Germany since the introduction of paediatric PCV13 vaccination [60, 67]. These data have led to a debate about whether there will be a reduced requirement for adult vaccination as the indirect effect of PCV13 increases. However, despite this potential herd effect, a significant burden of pneumococcal disease in adults remains and direct vaccination of adults is the optimal way to provide individual protection to those at risk and to significantly reduce the pneumococcal disease burden in the adult population.

There is also evidence that PCV10 has reduced the burden of pneumococcal diseases in children. Palmu et al. reported point estimates for PCV10 efficacy against vaccine-type IPD of 100 (95 % CI 83–100) for the 3 + 1 schedule and 92 (58–100) for the 2 + 1 schedule in a cluster-randomised, double-blind trial, involving Finnish children aged <19 months, vaccinated with PCV10 or hepatitis vaccines (control group) [68]. A PCV10 study in Canada has shown some evidence of reduced vaccine-type IPD in infants, with a reduction in IPD cases caused by the additional three PCV10 serotypes (1, 5 and 7F) observed following the introduction of PCV10 [69]. A reduction in hospitalisation rates due to pneumonia has also been reported in Brazil 1 year after the introduction of PCV10 into the NIP [70]. No available data as of July 2014 have been reported on the impact of PCV10 in non-vaccine eligible populations. PCV10 has been shown to be immunogenic in a number of primary and booster vaccination studies [71–75], and also to reduce vaccine-type pneumococcal carriage [76].

## PCV immunisation in other age groups

In Europe, PCV13 was licensed for use in individuals aged 6–49 years and for adults aged ≥50 years in 2011 [23]. Prior to this, PPV23 was the only pneumococcal vaccine licensed for active immunisation against vaccine-specific pneumococcal serotypes in adults in whom there is an increased risk of morbidity and mortality from pneumococcal diseases [77]. However, although PPV23 has been shown to reduce IPD in adults, data concerning the prevention of pneumococcal pneumonia or mortality are less clear [78]. Also, some limitations have been observed with this vaccine in immunodeficient patients, possibly associated with poor or absent immunogenicity [79]. In a randomised, placebo-controlled study (*n* = 1,392) conducted in Uganda, PPV23 was found to be ineffective in preventing a first episode of IPD in individuals with HIV (hazard ratio [HR] 1.47; 95 % CI 0.7–3.3) [80]. In contrast, in a more recent study conducted in Malawi, PCV7 was found to be effective in preventing vaccine-serotype IPD in individuals with HIV (*n* = 439) when compared with placebo (HR 0.26; 95 % CI 0.1–0.7) [81].

The immunogenicity and safety of PCV13 has been demonstrated in a clinical programme that supported licensure. This programme was designed to evaluate the functional immune response to PCV13 compared with PPV23 in two patient populations: adults 60–64 years of age who were naïve to PPV23 and those aged ≥70 years immunised with PPV23 at least 5 years before study enrolment [82, 83]. In both these populations, primary vaccination with PCV13 resulted in significantly higher anti-pneumococcal functional antibody responses (as assessed by opsonophagocytic activity [OPA] titres 1 month post-vaccination) than PPV23 for the majority of the PCV13 serotypes [82, 83]. Furthermore, unlike PPV23, PCV13 did not negatively affect the responses to a second dose of PCV13 administered 1 year later in the PPV23-preimmunised individuals aged ≥70 years [83]. An extension study conducted in the previously PPV23-naïve individuals also demonstrated that primary PCV13 vaccination can produce an immunological state, which allows recall anti-pneumococcal responses to subsequent vaccination with either PCV13 or PPV23. In contrast, primary PPV23 vaccination resulted in an immune state in which a subsequent PPV23 dose generally yielded inferior responses compared with the initial response [84]. In an analysis from three PCV13 clinical studies, the functional immune response to PCV13 in high-risk individuals with chronic medical conditions (diabetes mellitus or stable, chronic cardiovascular, pulmonary, liver or renal disease) was similar to that in non-high-risk individuals within these trials [85].

The immunogenicity of PCV13 has also been demonstrated in individuals aged 5–17 years. The immune response to PCV13 (as assessed by anti-pneumococcal IgG geometric mean concentrations) was shown to be non-inferior in children aged 5 to <10 years previously vaccinated with PCV7 compared with a PCV7/PCV13 post-toddler dose from a historical control study. The functional immunogenicity of PCV13 was also demonstrated to be non-inferior in PCV7-naïve children aged 10–17 years compared with that in those aged 5 to <10 years [86]. A similar bridging study was also conducted in the age group 18–49 years.

## What can we expect from pneumococcal vaccination?

**Fig. 3:**
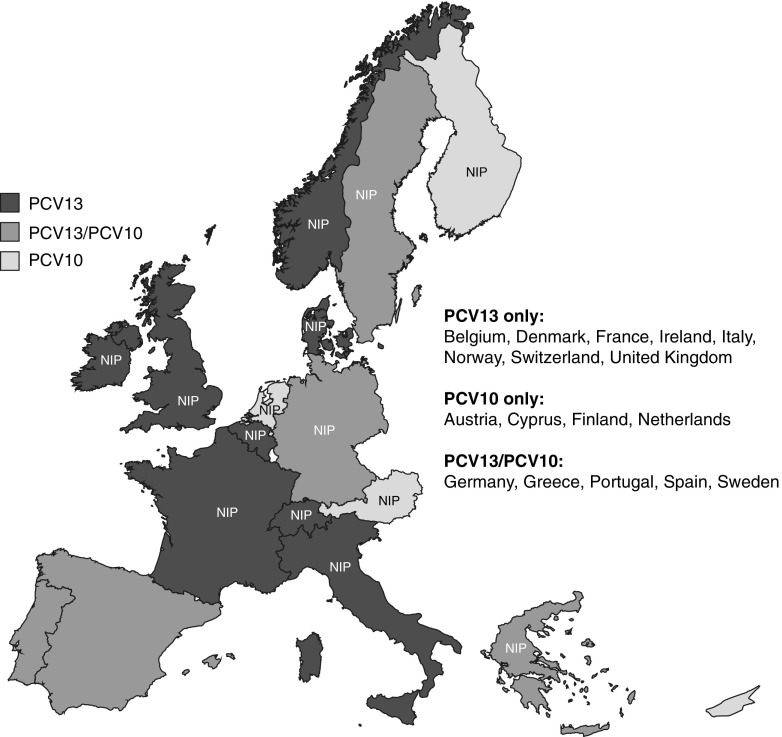
Pneumococcal conjugate vaccine usage in national childhood immunisation programmes in Western Europe. *PCV10*, 10-valent pneumococcal conjugate vaccine; *PCV13*, 13-valent pneumococcal conjugate vaccine

According to the WHO data, PCVs are used in 87 NIPs worldwide [21]. Within Western Europe, the use of specific PCVs in NIPs varies according to country (Fig. 3). In addition, national vaccination recommendations outside routine infant immunisation programmes differ among EU countries (Table 2). Some countries have age-based vaccination programmes, while others have risk-based programmes, and some countries have regional variations with respect to recommendations. There are also differences between scientific society guidelines. For example, in France and Spain, the French National Reference Center of Primary Immunodeficiencies (CEREDIH) [100] and the Spanish Society of Preventive Medicine, Public Health and Hygiene (SEMPSPH) [101], respectively, recommend PCV13 for immunocompromised patients. PCV13 is also recommended for renal failure and dialysis, and for smokers in Spain (SEMPSPH and Smoking Working Group of the Spanish Pulmonologist Society [SEPAR] recommendations, respectively) [101, 102]. In Germany, the Robert Koch Institute (RKI) and German Society for Hematology and Oncology (DGHO) guidelines recommend PCV13 followed by vaccination with PPV23 for asplenic patients [103, 104]. Within Italy, PCV13 is recommended in individuals of all ages within the ‘Vaccination calendar for life’ approved by the main public health, paediatric and general practitioner organisations [105]. These guidelines, however, may evolve as clinical experience with PCV13 increases, especially in relation to the more recent indications (adults ≥18 years of age and children/adolescents aged 6–17 years).

**Table 2:** National adult pneumococcal vaccination recommendations in Western Europe

Country (year)^a^	Vaccine	Pneumococcal vaccine recommendation	
		Age based	At-risk based, with definition of risk
Austria (2014) [87]	PCV13/PPV23	≥50 years	High-risk group (≥6 years): asplenia (anatomical, functional); chronic renal insufficiency; cochlear implant; complement and properdin deficiency; haematopoietic organ disorder; HIV; hypogammaglobulinaemia; immunodeficiency (congenital, acquired); liquor fistula; nephritic syndrome; nephrotic syndrome prior to immunosuppressive therapy; neurological disorder (in children); sickle cell anaemia; transplantation (organ, subsequent to stem cell transplantation) At-risk group (≥6 years): chronic cardiovascular disease (except hypertension); chronic respiratory disease; cirrhosis; diabetes; metabolic disease; neoplastic disease
Belgium (2013) [88]	PCV13/PPV23	≥65 years	High-risk groups (≥18 years): asplenia; autoimmune disease/immune-mediated inflammatory disease; cochlear implant; haematological cancer; HIV; immunodeficiency; organ transplantation Risk groups (≥50 years): alcoholism; chronic disease (heart, kidney, liver, respiratory); smoking
Denmark (2012) [89]	PCV13	≥65 years	At-risk group (any age): asplenia (functional); cochlear implant; CSF leak; HIV; history of IPD; lymphoma; organ transplantation; splenectomy (completed/planned) At-risk group (18–65 years): chronic disease (heart, kidney, liver, lung); diabetes mellitus
Finland (2013) [90]	PCV13	No	High risk (≥5 years): asplenia (functional, anatomical); cochlear implant; HIV; immunodeficiency (congenital, acquired); liquor fistula; lymphoma; multiple myeloma; nephrotic syndrome; patients treated with systemic corticosteroids or other immunosuppressants; transplantation (organ, tissue)
	PPV23	≥65 years	At risk or in permanent institutional care (≥5 years): chronic disease (cardiac, pulmonary); diabetes (type 1); hepatic insufficiency; patients treated with systemic corticosteroids or other immunosuppressants; renal insufficiency; transplantation (organ, tissue)
France (2013) [91]	PCV13	No	At-risk group (≥2 years): asplenia or hyposplenia; cancer treated by chemotherapy (solid tumour, haematological); cochlear implant or planned cochlear implant; HIV; immunodeficiency (congenital); immunosuppressive therapy, biotherapy, or corticotherapy for autoimmune disease or chronic inflammation; meningeal fistula; nephrotic syndrome; transplantation or waiting for transplantation (organ, haematopoietic stem cell)
	PPV23	No	At-risk group (≥5 years): asthma (severe with continuous treatment); chronic liver disease (alcoholic or non-alcoholic origin); chronic respiratory failure; COPD; cyanotic congenital heart disease; diabetes (not balanced by diet); emphysema; heart failure; kidney failure
Germany (2013) [92]	PCV13	≥60 years	At-risk group (≥2 years): asplenia; autoimmune disease; chronic disease (heart, kidney, respiratory); CSF leak; HIV; immunodeficiency (congenital or acquired); metabolic disease; neurologic disorder; transplantation (organ)
Germany (1982/1998) [92]	PPV23	≥60 years	At-risk group (≥5 years): asplenia; autoimmune disease; cancer (haematological, solid tumour); chronic disease (heart, kidney, liver, respiratory); CNS disease; CSF leak; HIV; immunodeficiency (congenital, acquired); metabolic disease; transplantation (organ)
Greece (2011) [93]	PCV13	>50 years	No
Ireland (2013) [94]	PPV23	≥65 years	High-risk group (18–64 years): asplenia, hyposplenia (including splenectomy, sickle cell disease, haemoglobinopathies and coeliac disease); cochlear implant (candidates, recipients); complement deficiency (particularly C1–C4); CSF leak (congenital, complicating skull fracture, neurosurgery); immunosuppressive conditions (e.g. some B- and T-cell disorders, HIV infection, leukaemia, lymphoma) and those receiving immunosuppressive therapies; intracranial shunt; post-haematopoietic stem cell transplant; solid organ transplant At-risk group (18–64 years): chronic heart, lung or liver disease; chronic renal disease or nephrotic syndrome; diabetes mellitus requiring insulin or oral hypoglycaemic drugs; individuals with occupational exposure to metal fumes (i.e. welders)
Luxembourg (2008) [95]	PPV23	>60 years	At risk or in permanent institutional care (≥18 years): alcoholism; asplenia; chronic disease (cardiovascular, renal, respiratory); cochlear implant; CSF leak; diabetes; HIV; liquor fistula; liver cirrhosis; lymphoma; multiple myeloma; nephrotic syndrome; sickle cell disease; transplantation (organ)
Norway (2013) [96, 97]	PCV13	No	At-risk groups (all ages): asplenia; HIV; stem cell transplantation Also, considered for following groups after collective evaluation of risk: B-cell deficiency; cancer (haematological); cochlear implant; CSF leak; transplantation (organ)
	PPV23	≥65 years	At-risk groups (all ages): asplenia; B-cell deficiency; cancer (haematological); cochlear implant; CSF leak; HIV; transplantation (organ, bone marrow)
Sweden (1994) [98]	PPV23	≥65 years	At-risk group (≥2 years): agammaglobulinaemia; alcoholism; asplenia; asthma; autoimmune disease; cancer (haematological, solid tumour); chronic disease (heart, kidney, liver, respiratory); cyanotic heart disease; CNS disease; CSF leak; haemodynamically significant residual lesion after surgery; haemodynamic respiratory insufficiency; history of IPD; HIV; immunodeficiency (primary); intracranial shunt; metabolic disease; SCID; sickle cell disease and other haemoglobinopathies; transplantation (organ)
United Kingdom (2013) [99]	PCV13	No	At-risk group (≥5 years): severely immunocompromised: genetic disorders severely affecting the immune system (e.g. IRAK-4, NEMO, complement deficiency); leukaemia (acute, chronic); multiple myeloma; transplantation (bone marrow)
United Kingdom (1992/2003) [99]	PPV23	≥65 years	At-risk group (≥2 years): asplenia; asthma (only if high-dose systemic steroids); cancer (haematological, solid tumour); chronic disease (heart, kidney, liver, respiratory); cochlear implant; CSF leak; diabetes (excludes diet controlled); HIV; immunosuppression; sickle cell disease; transplantation (organ)

CSF: cerebrospinal fluid; CNS: central nervous system; COPD: chronic obstructive pulmonary disease; HIV: human immunodeficiency virus; IPD: invasive pneumococcal disease; PCV: pneumococcal conjugate vaccine; PPV: pneumococcal polysaccharide vaccine; SCID: severe combined immunodeficiency

^a^Date of implementation of recommendation

The WHO has published guidance for the introduction of new vaccines [106]. Cost-effectiveness, as well as disease burden, efficacy, safety and quality are recommended as key elements that should be considered as part of this process. In Italy, the clinical and economic impact of an adult pneumococcal vaccination programme has been assessed using an ad hoc population model and paediatric efficacy data [107]. This analysis found that, as a consequence of avoided pneumococcal infections, age-based PCV13 immunisation in elderly individuals (aged ≥65 years) resulted in savings ranging from 7 to 19 million Euros, depending on the vaccination strategy. An adult pneumococcal vaccination programme was, therefore, considered to be cost-effective from the payer perspective. Similarly, a German cost-effectiveness analysis, which also utilised paediatric data, indicated that adult PCV13 vaccination would provide substantial health and economic benefits relative to PPV23 or no vaccination [108]. However, these findings will need to be verified with adult efficacy data, when available. The efficacy of PCV13 in the prevention of a first episode of vaccine-serotype-specific pneumococcal CAP was studied in the Community-Acquired Pneumonia Immunization Trial in Adults (CAPiTA) [109] that was recently completed. This randomised placebo-controlled trial [110] involved a pneumococcal vaccination-naïve population of 85,000 community-dwelling adults ≥65 years of age from the Netherlands.

Given both data from clinical trials and our experience of paediatric PCV13 vaccination, a beneficial effect could be expected with adult vaccination. A global literature review demonstrated reductions in vaccine-type and all-type IPD in vaccine-eligible children as well as age groups that were not eligible for vaccination following PCV7 introduction [111]. Within the 18 studies assessing the impact of PCV7 in vaccine-eligible children in the post-vaccination period, the median rate of reductions in vaccine-type IPD incidence was 90.1 % (range 39.9–99.1 %). Another literature review reported reductions in hospitalisation rates associated with pneumococcal pneumonia, ranging from 57 to 71 %, in children aged <2 years, as well as a reduction in mortality associated with all-type IPD in children [112]. There is also emerging, global evidence of a reduced burden of pneumococcal diseases (IPD, AOM and meningitis) in children following the introduction of PCV13. In addition to reductions in IPD cases within the UK and Germany (mentioned above), there have also been reports of reductions in the incidence of vaccine-type IPD in the USA and Spain [113, 114], as well as reductions in meningitis cases in Greece [115] and vaccine-type AOM cases in the USA and Spain [116, 117].

## Conclusion

Given the high burden of pneumococcal diseases, pneumococcal vaccination is a key element of global disease prevention. Conjugate vaccines elicit a qualitatively and quantitatively higher level of immune response than polysaccharide vaccines, and, thus, provide greater immunity in children <2 years of age and in those with compromised immunity than polysaccharide vaccines. 13-valent pneumococcal conjugate vaccine (PCV13) expectations in the more recently indicated populations (adults aged ≥50 years and children and adolescents) can be built on the heritage of PCV7 effectiveness data in children, as well as emerging impact data for PCV13. The Community-Acquired Pneumonia Immunization Trial in Adults (CAPiTA) trial should provide more definitive data on the role of adult PCV13 vaccination in preventing vaccine-serotype-specific pneumococcal community-acquired pneumonia (CAP). Continued surveillance will be important to determine the impact of PCV13 on pneumococcal diseases in these age groups and also to monitor the evolution of causative serotypes.

## References

[1] Butler JC, Schuchat A (1999). Epidemiology of pneumococcal infections in the elderly. Drugs Aging.

[2] Torné AN, Dias JG, Quinten C, Hruba F, Busana MC, Lopalco PL, Gauci AJ, Pastore-Celentano L, ECDC country experts for pneumococcal disease (2014). European enhanced surveillance of invasive pneumococcal disease in 2010: data from 26 European countries in the post-heptavalent conjugate vaccine era. Vaccine.

[3] Rueda AM, Serpa JA, Matloobi M, Mushtaq M, Musher DM (2010). The spectrum of invasive pneumococcal disease at an adult tertiary care hospital in the early 21st century. Medicine (Baltimore).

[4] Welte T (2012). How can we reduce the mortality of invasive pneumococcal disease?. Eur Respir Rev.

[5] O’Brien KL, Wolfson LJ, Watt JP, Henkle E, Deloria-Knoll M, McCall N, Lee E, Mulholland K, Levine OS, Cherian T, Hib and Pneumococcal Global Burden of Disease Study Team (2009). Burden of disease caused by Streptococcus pneumoniae in children younger than 5 years: global estimates. Lancet.

[6] Jansen AG, Rodenburg GD, de Greeff SC, Hak E, Veenhoven RH, Spanjaard L, Schouls LM, Sanders EA, van der Ende A (2009) Invasive pneumococcal disease in the Netherlands: syndromes, outcome and potential vaccine benefits. Vaccine 27:2394–2401 10.1016/j.vaccine.2009.01.127 19428856

[7] Torres A, Rodríguez-Créixems M, Grau I, Molinos L, Llinares P, De la Cruz JL, Rajas O, Alfageme I, Salavert M, Fenoll A, Liñares J, Cifuentes I (2013) Underlying clinical conditions and invasive pneumococcal disease (IPD) in adults in Spain (ODIN study, 2010–2012). Abstract 5051. European Respiratory Society (ERS) Annual Congress 2013, Barcelona, Spain, 7–11 September 2013. Available online at: http://www.ers-education.org/events/annual-congress.aspx?idParent=125517. Accessed 12 August 2014

[8] van Hoek AJ, Andrews N, Waight PA, Stowe J, Gates P, George R, Miller E (2012). The effect of underlying clinical conditions on the risk of developing invasive pneumococcal disease in England. J Infect.

[9] Whitney CG, Farley MM, Hadler J, Harrison LH, Bennett NM, Lynfield R, Reingold A, Cieslak PR, Pilishvili T, Jackson D, Facklam RR, Jorgensen JH, Schuchat A, Active Bacterial Core Surveillance of the Emerging Infections Program Network (2003). Decline in invasive pneumococcal disease after the introduction of protein–polysaccharide conjugate vaccine. N Engl J Med.

[10] Poehling KA, Talbot TR, Griffin MR, Craig AS, Whitney CG, Zell E, Lexau CA, Thomas AR, Harrison LH, Reingold AL, Hadler JL, Farley MM, Anderson BJ, Schaffner W (2006). Invasive pneumococcal disease among infants before and after introduction of pneumococcal conjugate vaccine. JAMA.

[11] Isaacman DJ, McIntosh ED, Reinert RR (2010). Burden of invasive pneumococcal disease and serotype distribution among Streptococcus pneumoniae isolates in young children in Europe: impact of the 7-valent pneumococcal conjugate vaccine and considerations for future conjugate vaccines. Int J Infect Dis.

[12] Rose MA, Christopoulou D, Myint TT, de Schutter I (2014). The burden of invasive pneumococcal disease in children with underlying risk factors in North America and Europe. Int J Clin Pract.

[13] Nuorti JP (2000) Epidemiology of invasive pneumococcal disease in adults: implications for prevention. National Public Health Institute, Helsinki, Finland. Available online at: http://ethesis.helsinki.fi/julkaisut/laa/kliin/vk/nuorti/epidemio.pdf. Accessed 12 August 2014

[14] Advisory Committee on Immunization Practices (2009). Recommended adult immunization schedule: United States, 2009. Ann Intern Med.

[15] Rahier JF, Moutschen M, Van Gompel A, Van Ranst M, Louis E, Segaert S, Masson P, De Keyser F (2010). Vaccinations in patients with immune-mediated inflammatory diseases. Rheumatology (Oxford).

[16] Gil-Prieto R, García-García L, Alvaro-Meca A, Méndez C, García A, de Miguel AG (2011). The burden of hospitalisations for community-acquired pneumonia (CAP) and pneumococcal pneumonia in adults in Spain (2003–2007). Vaccine.

[17] Welte T, Torres A, Nathwani D (2012). Clinical and economic burden of community-acquired pneumonia among adults in Europe. Thorax.

[18] Torres A, Peetermans WE, Viegi G, Blasi F (2013). Risk factors for community-acquired pneumonia in adults in Europe: a literature review. Thorax.

[19] Wright AE, Morgan WP, Colebrook L, Dodgson RW (1914). Observations on prophylactic inoculation against pneumococcus infections, and on the results which have been achieved by it. Lancet.

[20] Reinert RR, Paradiso P, Fritzell B (2010). Advances in pneumococcal vaccines: the 13-valent pneumococcal conjugate vaccine received market authorization in Europe. Expert Rev Vaccines.

[21] Centers for Disease Control and Prevention (CDC) (2013). Progress in introduction of pneumococcal conjugate vaccine—worldwide, 2000–2012. MMWR Morb Mortal Wkly Rep.

[22] GlaxoSmithKline Biologicals s.a. (2014) Synoflorix: summary of product characteristics. European Medicines Agency (EMA). Available online at: http://www.ema.europa.eu/docs/en_GB/document_library/EPAR_-_Product_Information/human/000973/WC500054346.pdf. Accessed 12 August 2014

[23] electronic Medicines Compendium (eMC) (2014) Prevenar 13 suspension for injection. eMC. Available online at: http://www.medicines.org.uk/emc/medicine/22689/SPC/Prevenar+13+suspension+for+injection/. Accessed 12 August 2014

[24] Blanchard-Rohner G, Pollard AJ (2011). Long-term protection after immunization with protein–polysaccharide conjugate vaccines in infancy. Expert Rev Vaccines.

[25] Coutinho A, Möller G (1973). B cell mitogenic properties of thymus-independent antigens. Nat New Biol.

[26] Barrett DJ (1985). Human immune responses to polysaccharide antigens: an analysis of bacterial polysaccharide vaccines in infants. Adv Pediatr.

[27] Barrett DJ, Ayoub EM (1986). IgG2 subclass restriction of antibody to pneumococcal polysaccharides. Clin Exp Immunol.

[28] Toellner KM, Jenkinson WE, Taylor DR, Khan M, Sze DM, Sansom DM, Vinuesa CG, MacLennan IC (2002). Low-level hypermutation in T cell-independent germinal centers compared with high mutation rates associated with T cell-dependent germinal centers. J Exp Med.

[29] Taillardet M, Haffar G, Mondière P, Asensio MJ, Gheit H, Burdin N, Defrance T, Genestier L (2009). The thymus-independent immunity conferred by a pneumococcal polysaccharide is mediated by long-lived plasma cells. Blood.

[30] Obukhanych TV, Nussenzweig MC (2006). T-independent type II immune responses generate memory B cells. J Exp Med.

[31] Martin F, Oliver AM, Kearney JF (2001). Marginal zone and B1 B cells unite in the early response against T-independent blood-borne particulate antigens. Immunity.

[32] Birjandi SZ, Ippolito JA, Ramadorai AK, Witte PL (2011). Alterations in marginal zone macrophages and marginal zone B cells in old mice. J Immunol.

[33] Tortola L, Yadava K, Bachmann MF, Müller C, Kisielow J, Kopf M (2010). IL-21 induces death of marginal zone B cells during chronic inflammation. Blood.

[34] Kruetzmann S, Rosado MM, Weber H, Germing U, Tournilhac O, Peter HH, Berner R, Peters A, Boehm T, Plebani A, Quinti I, Carsetti R (2003). Human immunoglobulin M memory B cells controlling Streptococcus pneumoniae infections are generated in the spleen. J Exp Med.

[35] Kruschinski C, Zidan M, Debertin AS, von Hörsten S, Pabst R (2004). Age-dependent development of the splenic marginal zone in human infants is associated with different causes of death. Hum Pathol.

[36] Brodeur PH, Wortis HH (1980). Regulation of thymus-independent responses: unresponsiveness to a second challenge of TNP-Ficoll is mediated by hapten-specific antibodies. J Immunol.

[37] Pollard AJ, Perrett KP, Beverley PC (2009). Maintaining protection against invasive bacteria with protein–polysaccharide conjugate vaccines. Nat Rev Immunol.

[38] Brynjolfsson SF, Henneken M, Bjarnarson SP, Mori E, Del Giudice G, Jonsdottir I (2012). Hyporesponsiveness following booster immunization with bacterial polysaccharides is caused by apoptosis of memory B cells. J Infect Dis.

[39] Kanswal S, Katsenelson N, Allman W, Uslu K, Blake MS, Akkoyunlu M (2011). Suppressive effect of bacterial polysaccharides on BAFF system is responsible for their poor immunogenicity. J Immunol.

[40] Wu ZQ, Vos Q, Shen Y, Lees A, Wilson SR, Briles DE, Gause WC, Mond JJ, Snapper CM (1999). In vivo polysaccharide-specific IgG isotype responses to intact Streptococcus pneumoniae are T cell dependent and require CD40- and B7-ligand interactions. J Immunol.

[41] MacLennan IC (1994). Germinal centers. Annu Rev Immunol.

[42] Avci FY, Li X, Tsuji M, Kasper DL (2011). A mechanism for glycoconjugate vaccine activation of the adaptive immune system and its implications for vaccine design. Nat Med.

[43] Centers for Disease Control and Prevention (CDC) (2008). Invasive pneumococcal disease in children 5 years after conjugate vaccine introduction—eight states, 1998–2005. MMWR Morb Mortal Wkly Rep.

[44] Roche PW, Krause V, Cook H, Barralet J, Coleman D, Sweeny A, Fielding J, Giele C, Gilmour R, Holland R, Kampen R, Brown M, Gilbert L, Hogg G, Murphy D, Enhanced Invasive Pneumococcal Disease Surveillance Working Group, Pneumococcal Working Party of the Communicable Diseases Network Australia (2008). Invasive pneumococcal disease in Australia, 2006. Commun Dis Intell Q Rep.

[45] Kellner JD, Church DL, MacDonald J, Tyrrell GJ, Scheifele D (2005). Progress in the prevention of pneumococcal infection. CMAJ.

[46] Dubos F, Marechal I, Husson MO, Courouble C, Aurel M, Martinot A, Hospital Network for Evaluating the Management of Common Childhood Diseases (2007). Decline in pneumococcal meningitis after the introduction of the heptavalent-pneumococcal conjugate vaccine in northern France. Arch Dis Child.

[47] Rückinger S, van der Linden M, Reinert RR, von Kries R, Burckhardt F, Siedler A (2009). Reduction in the incidence of invasive pneumococcal disease after general vaccination with 7-valent pneumococcal conjugate vaccine in Germany. Vaccine.

[48] Vestrheim DF, Løvoll O, Aaberge IS, Caugant DA, Høiby EA, Bakke H, Bergsaker MR (2008). Effectiveness of a 2+1 dose schedule pneumococcal conjugate vaccination programme on invasive pneumococcal disease among children in Norway. Vaccine.

[49] Pérez-Trallero E, Marimon JM, Ercibengoa M, Vicente D, Pérez-Yarza EG (2009). Invasive Streptococcus pneumoniae infections in children and older adults in the north of Spain before and after the introduction of the heptavalent pneumococcal conjugate vaccine. Eur J Clin Microbiol Infect Dis.

[50] Grijalva CG, Nuorti JP, Arbogast PG, Martin SW, Edwards KM, Griffin MR (2007). Decline in pneumonia admissions after routine childhood immunisation with pneumococcal conjugate vaccine in the USA: a time-series analysis. Lancet.

[51] Patrzałek M, Albrecht P, Sobczynski M (2010). Significant decline in pneumonia admission rate after the introduction of routine 2+1 dose schedule heptavalent pneumococcal conjugate vaccine (PCV7) in children under 5 years of age in Kielce, Poland. Eur J Clin Microbiol Infect Dis.

[52] Poehling KA, Szilagyi PG, Grijalva CG, Martin SW, LaFleur B, Mitchel E, Barth RD, Nuorti JP, Griffin MR (2007). Reduction of frequent otitis media and pressure-equalizing tube insertions in children after introduction of pneumococcal conjugate vaccine. Pediatrics.

[53] Zhou F, Shefer A, Kong Y, Nuorti JP (2008). Trends in acute otitis media-related health care utilization by privately insured young children in the United States, 1997–2004. Pediatrics.

[54] Kyaw MH, Lynfield R, Schaffner W, Craig AS, Hadler J, Reingold A, Thomas AR, Harrison LH, Bennett NM, Farley MM, Facklam RR, Jorgensen JH, Besser J, Zell ER, Schuchat A, Whitney CG, Active Bacterial Core Surveillance of the Emerging Infections Program Network (2006). Effect of introduction of the pneumococcal conjugate vaccine on drug-resistant Streptococcus pneumoniae. N Engl J Med.

[55] O’Brien KL, Millar EV, Zell ER, Bronsdon M, Weatherholtz R, Reid R, Becenti J, Kvamme S, Whitney CG, Santosham M (2007). Effect of pneumococcal conjugate vaccine on nasopharyngeal colonization among immunized and unimmunized children in a community-randomized trial. J Infect Dis.

[56] Grivea IN, Panagiotou M, Tsantouli AG, Syrogiannopoulos GA (2008). Impact of heptavalent pneumococcal conjugate vaccine on nasopharyngeal carriage of penicillin-resistant Streptococcus pneumoniae among day-care center attendees in central Greece. Pediatr Infect Dis J.

[57] Davis SM, Deloria-Knoll M, Kassa HT, O’Brien KL (2013). Impact of pneumococcal conjugate vaccines on nasopharyngeal carriage and invasive disease among unvaccinated people: review of evidence on indirect effects. Vaccine.

[58] Barricarte A, Castilla J, Gil-Setas A, Torroba L, Navarro-Alonso JA, Irisarri F, Arriazu M (2007). Effectiveness of the 7-valent pneumococcal conjugate vaccine: a population-based case–control study. Clin Infect Dis.

[59] Bekri H, Cohen R, Varon E, Madhi F, Gire R, Guillot F, Delacourt C (2007). Streptococcus pneumoniae serotypes involved in children with pleural empyemas in France. Arch Pediatr.

[60] Public Health England (2013) Current Epidemiology of Invasive Pneumococcal Disease (IPD). Public Health England. Available online at: http://www.hpa.org.uk/Topics/InfectiousDiseases/InfectionsAZ/Pneumococcal/EpidemiologicalDataPneumococcal/CurrentEpidemiologyPneumococcal/. Accessed 14 May 2013

[61] van der Linden M, von Kries R, Imöhl M (2012) Effects of three years of immunization with higher valent pneumococcal conjugate vaccines on serotype distributions among reported IPD cases in German children and adults. Presented at the 52nd ICAAC, September 9–12, 2012, San Francisco, CA, USA. Available online at: http://www.abstractsonline.com/Plan/ViewAbstract.aspx?sKey=becf1b8c-545a-4792-ac02-4a9cb31fdf7b&cKey=bb809851-e003-4b87-8957-043e6ed60add&mKey=%7b6B114A1D-85A4-4054-A83B-04D8B9B8749F%7d. Accessed 12 August 2014

[62] Félix S, Valente C, Tavares DA, Nunes S, Brito-Avô A, de Lencastre H, Sá-Leão R (2012) Temporal trends of serotypes included in the novel 13-valent pneumococcal conjugate vaccine (PCV13) among young children from Portugal. Presented at the 8th International Symposium on Pneumococci and Pneumococcal Diseases, Iguaçu Falls, Brazil, March 11–15, 2012. Available online at: http://www2.kenes.com/ISPPD/Scientific/Documents/FinalAbstractbook.pdf. Accessed 12 August 2014

[63] Cohen R, Levy C, Bingen E, Bonnet E, Koskas M, Attal S, Nave I, Fritzell B, Varon E (2011) Impact of 13-valent pneumococcal conjugate vaccine (PCV13) on nasopharyngeal (NP) flora in children with acute otitis media (AOM). Presented at the 51st ICAAC, September 17–20, 2011, Chicago, USA. Available online at: http://www.abstractsonline.com/Plan/ViewAbstract.aspx?sKey=b0b69d8d-27d1-43f4-8f4e-9b651a2028f3&cKey=a8db6616-5ef8-484f-b65e-4e1d48fcfdac&mKey=0C918954-D607-46A7-8073-44F4B537A439. Accessed 12 August 2014

[64] Nuorti JP, Butler JC, Farley MM, Harrison LH, McGeer A, Kolczak MS, Breiman RF (2000). Cigarette smoking and invasive pneumococcal disease. Active Bacterial Core Surveillance Team. N Engl J Med.

[65] Steens A, Bergsaker MA, Aaberge IS, Rønning K, Vestrheim DF (2013). Prompt effect of replacing the 7-valent pneumococcal conjugate vaccine with the 13-valent vaccine on the epidemiology of invasive pneumococcal disease in Norway. Vaccine.

[66] Norwegian Institute of Public Health (2013) Vaccination statistics for 2012—95 per cent of 9-year-olds are vaccinated. Norwegian Institute of Public Health. Available online at: http://www.fhi.no/artikler/?id=105667. Accessed 12 August 2014

[67] van der Linden M, Imohl M (2013) Serotype distribution and vaccine coverage among pneumococcal disease in adults in Germany [Poster eP737]. Presented at the 23rd European Congress of Clinical Microbiology and Infectious Diseases (ECCMID), Berlin, Germany, 27–30 April 2013. Available online at: https://www.escmid.org/escmid_library/online_lecture_library/?search=1&current_page=1&search_term=Linden&entrytype%5B%5D=18&entrytype%5B%5D=20&entrytype%5B%5D=17&entrytype%5B%5D=19&entrytitle%5B%5D=4534. Accessed 12 August 2014

[68] Palmu AA, Jokinen J, Borys D, Nieminen H, Ruokokoski E, Siira L, Puumalainen T, Lommel P, Hezareh M, Moreira M, Schuerman L, Kilpi TM (2013). Effectiveness of the ten-valent pneumococcal Haemophilus influenzae protein D conjugate vaccine (PHiD-CV10) against invasive pneumococcal disease: a cluster randomised trial. Lancet.

[69] Lim GH, Wormsbecker AE, McGeer A, Pillai DR, Gubbay JB, Rudnick W, Low DE, Green K, Crowcroft NS, Deeks SL (2013). Have changing pneumococcal vaccination programmes impacted disease in Ontario?. Vaccine.

[70] Afonso ET, Minamisava R, Bierrenbach AL, Escalante JJ, Alencar AP, Domingues CM, Morais-Neto OL, Toscano CM, Andrade AL (2013). Effect of 10-valent pneumococcal vaccine on pneumonia among children, Brazil. Emerg Infect Dis.

[71] van den Bergh MR, Spijkerman J, François N, Swinnen K, Borys D, Schuerman L, Veenhoven RH, Sanders EA (2011). Immunogenicity, safety, and reactogenicity of the 10-valent pneumococcal nontypeable Haemophilus influenzae protein D conjugate vaccine and DTPa-IPV-Hib when coadministered as a 3-dose primary vaccination schedule in The Netherlands: a randomized controlled trial. Pediatr Infect Dis J.

[72] Vesikari T, Wysocki J, Chevallier B, Karvonen A, Czajka H, Arsène JP, Lommel P, Dieussaert I, Schuerman L (2009). Immunogenicity of the 10-valent pneumococcal non-typeable Haemophilus influenzae protein D conjugate vaccine (PHiD-CV) compared to the licensed 7vCRM vaccine. Pediatr Infect Dis J.

[73] Bermal N, Szenborn L, Chrobot A, Alberto E, Lommel P, Gatchalian S, Dieussaert I, Schuerman L (2009). The 10-valent pneumococcal non-typeable Haemophilus influenzae protein D conjugate vaccine (PHiD-CV) coadministered with DTPw-HBV/Hib and poliovirus vaccines: assessment of immunogenicity. Pediatr Infect Dis J.

[74] Dicko A, Odusanya OO, Diallo AI, Santara G, Barry A, Dolo A, Diallo A, Kuyinu YA, Kehinde OA, François N, Borys D, Yarzabal JP, Moreira M, Schuerman L (2011). Primary vaccination with the 10-valent pneumococcal non-typeable Haemophilus influenzae protein D conjugate vaccine (PHiD-CV) in infants in Mali and Nigeria: a randomized controlled trial. BMC Public Health.

[75] Ruiz-Palacios GM, Guerrero ML, Hernández-Delgado L, Lavalle-Villalobos A, Casas-Muñoz A, Cervantes-Apolinar Y, Moreira M, Schuerman L (2011). Immunogenicity, reactogenicity and safety of the 10-valent pneumococcal nontypeable Haemophilus influenzae protein D conjugate vaccine (PHiD-CV) in Mexican infants. Hum Vaccine.

[76] Hammitt LL, Ojal J, Bashraheil M, Morpeth SC, Karani A, Habib A, Borys D, Goldblatt D, Scott JA (2014). Immunogenicity, impact on carriage and reactogenicity of 10-valent pneumococcal non-typeable Haemophilus influenzae protein D conjugate vaccine in Kenyan children aged 1–4 years: a randomized controlled trial. PLoS One.

[77] Sanofi Pasteur MSD (2014) Pneumovax 23: summary of product characteristics. eMC. Available online at: http://www.medicines.org.uk/emc/medicine/1446. Accessed 12 August 2014

[78] Moberley S, Holden J, Tatham DP, Andrews RM (2013) Vaccines for preventing pneumococcal infection in adults. Cochrane Database Syst Rev 1:CD000422 10.1002/14651858.CD000422.pub3 PMC7045867 23440780

[79] World Health Organization (WHO) (2012). Pneumococcal vaccines WHO position paper—2012. Wkly Epidemiol Rec.

[80] French N, Nakiyingi J, Carpenter LM, Lugada E, Watera C, Moi K, Moore M, Antvelink D, Mulder D, Janoff EN, Whitworth J, Gilks CF (2000). 23-valent pneumococcal polysaccharide vaccine in HIV-1-infected Ugandan adults: double-blind, randomised and placebo controlled trial. Lancet.

[81] French N, Gordon SB, Mwalukomo T, White SA, Mwafulirwa G, Longwe H, Mwaiponya M, Zijlstra EE, Molyneux ME, Gilks CF (2010). A trial of a 7-valent pneumococcal conjugate vaccine in HIV-infected adults. N Engl J Med.

[82] Jackson LA, Gurtman A, van Cleeff M, Jansen KU, Jayawardene D, Devlin C, Scott DA, Emini EA, Gruber WC, Schmoele-Thoma B (2013). Immunogenicity and safety of a 13-valent pneumococcal conjugate vaccine compared to a 23-valent pneumococcal polysaccharide vaccine in pneumococcal vaccine-naive adults. Vaccine.

[83] Jackson LA, Gurtman A, Rice K, Pauksens K, Greenberg RN, Jones TR, Scott DA, Emini EA, Gruber WC, Schmoele-Thoma B (2013). Immunogenicity and safety of a 13-valent pneumococcal conjugate vaccine in adults 70 years of age and older previously vaccinated with 23-valent pneumococcal polysaccharide vaccine. Vaccine.

[84] Jackson LA, Gurtman A, van Cleeff M, Frenck RW, Treanor J, Jansen KU, Scott DA, Emini EA, Gruber WC, Schmoele-Thoma B (2013). Influence of initial vaccination with 13-valent pneumococcal conjugate vaccine or 23-valent pneumococcal polysaccharide vaccine on anti-pneumococcal responses following subsequent pneumococcal vaccination in adults 50 years and older. Vaccine.

[85] Schmoele-Thoma A, Jackson LA, Greenberg RN, Frenck R, Gurtman A, Jayawardene D, Emini EA, Gruber W, Scott, DA (2012) The immunogenicity of Prevnar 13 in immunocompetent older adults with stable high risk conditions is comparable to that in healthy older adults. Presented at IDWeek 2012, San Diego, CA, October 17–21, 2012. Available online at: https://idsa.confex.com/idsa/2012/webprogram/Paper36522.html. Accessed 12 August 2014

[86] Frenck R Jr, Thompson A, Senders S, Patterson S, Devlin C, Jansen K, Gruber WC, Emini EA, Scott DA, Gurtman A (2012) 13-valent pneumococcal conjugate vaccine in older children and teens either previously immunized with or naïve to 7-valent pneumococcal conjugate vaccine. Presented at the 8th International Symposium on Pneumococci and Pneumococcal Diseases, Iguaçu Falls, Brazil, March 11–15, 2012. Available online at: http://www2.kenes.com/ISPPD/Scientific/Documents/FinalAbstractbook.pdf. Accessed 12 August 2014

[87] Bundesministerium für Gesundheit (2014) Österreichischer Impfplan 2014. Bundesministerium für Gesundheit. Available online at: http://bmg.gv.at/home/Schwerpunkte/Praevention/Impfen/Oesterreichischer_Impfplan_2014. Accessed 12 August 2014

[88] Conseil Supérieur de la Santé (2013) Vaccination antipneumococcique: vaccination de l’adulte (révision 13). Conseil Supérieur de la Santé. Available online at: http://www.health.belgium.be/filestore/19086989_FR/vaccination%20fiche%20contre%20pneumoccoque.pdf. Accessed 12 August 2014

[89] Statens Serum Institut (2012) Pneumococcal vaccination of persons at increased risk of invasive pneumococcal disease. EPI-NEWS. Available online at: http://www.ssi.dk/English/News/EPI-NEWS/2012/No%2051b%20-%202012.aspx. Accessed 12 August 2014

[90] Terveyden ja hyvinvoinnin laitos (2013) Pneumokokkirokotukset. Rokottajan käsikirja. Available online at: http://www.thl.fi/fi_FI/web/rokottajankasikirja-fi/pneumokokkirokotukset. Accessed 12 August 2014

[91] Haut Conseil de la Santé Publique (2013) Infections invasives à pneumocoque : recommandations vaccinales pour les personnes à risque. Haut Conseil de la Santé Publique. Available online at: http://www.hcsp.fr/Explore.cgi/avisrapportsdomaine?clefr=355. Accessed 12 August 2014

[92] Richtlinie Schutzimpfungs-Richtlinie Stand (2013) 20. Dezember 2013 des Gemeinsamen Bundesausschusses über Schutzimpfungen nach § 20d Abs. 1 SGB V. Gemeinersamer Bundesausschuss. Available online at: https://www.g-ba.de/downloads/62-492-813/SI-RL_2013-10-01.pdf. Accessed 12 August 2014

[93] Διεύθυνση Δημόσιας Υγιεινής του Υπουργείου Υγείας και Κοινωνικής Αλληλεγγύης. Εθνική Επιτροπή Εμβολιασμών. Πρόγραμμα Εμβολιασμών Ενηλίκων (Αθήνα 21/12/2011, Αρ Πρωτ. Υ1/Γ.Π.οικ.140958) Available: http://static.diavgeia.gov.gr/doc/45%CE%A8%CE%A8%CE%98-2%CE%98%CE%9C. Accessed 12 August 2014

[94] National Immunisation Advisory Committee of the Royal College of Physicians of Ireland (2013) Immunisation Guidelines for Ireland, 2013 Edition (updated June 2014). Chapter 16: pneumococcal infection. Health Service Executive. Available online at: http://www.immunisation.ie/en/HealthcareProfessionals/ImmunisationGuidelines/. Accessed 12 August 2014

[95] Conseil Superieur D’hydiene (2008) Recommandations pour la vaccination contre le pneumocoque par le vaccin 23valent. Grand Duché de Luxembourg. Available online at: http://www.sante.public.lu/fr/recommandations/conseil-maladies-infectieuses/pneumonie/recommandations-vaccinations-2008/pneumonie-recommandations-vaccinatin-pneumocoque-23valent-2008.pdf. Accessed 12 August 2014

[96] Nasjonalt folkehelseinstitutt (2013) Anbefalinger for bruk av pneumokokkvaksine utenfor barnevaksinasjonsprogram i Norge. Nasjonalt folkehelseinstitutt. Available online at: http://www.fhi.no/dokumenter/14a5077fef.pdf. Accessed 12 August 2014

[97] Helse- og omsorgsdepartementet (2007) Forskrift om stønad til dekning av utgifter til viktige legemidler mv. (blåreseptforskriften). Lovdata. Available online at: http://www.lovdata.no/for/sf/ho/xo-20070628-0814.html. Accessed 12 August 2014

[98] Socialstyrelsen (1994) SOSFS 1994:26: Socialstyrelsens allmänna råd; Vaccination mot pneumokocker. Socialstyrelsen. Available online at: http://www.socialstyrelsen.se/publikationer1994/1994-10-26. Accessed 12 August 2014

[99] Public Health England (2013) Pneumococcal: the green book, chapter 25. gov.uk. Available online at: https://www.gov.uk/government/publications/pneumococcal-the-green-book-chapter-25. Accessed 12 August 2014

[100] Obenga G, Mahlaoul N, Launay O (2009) Recommandations vaccinales chez les patients atteints de déficit immunitaire héréditaire. CEREDIH. Available online at: http://www.ceredih.fr/documents/Recos_Vaccins_20091113.pdf. Accessed 12 August 2014

[101] Dominguez V, Arrazola P, Campins M, Chamorro J, de Diego J, Fenoli A, Gil A, Mollar J, Quintas C, Torres Lana A (2012) Recomendaciones de vacunación antineumocócica en el adulto por indicación médica. SEMPSPH. Available online at: http://www.sempsph.com/images/stories/recursos/pdf/protocolos/2012/Recom_Vac_Antineumococica_SEMPSPH.pdf. Accessed 12 August 2014

[102] Jiménez R, Solano R, Riesco M, Altet G, Signes-Costa M, Lorza B, de Granda O, Ramos P, de Higes M, Plaza V, Celdrán G, Martínez M, Santacruz S (2012) Recomendaciones para la vacunación neumocócica en fumadores. Prev Tab. Available online at: http://issuu.com/separ/docs/prev_tab_14-4?e=3049452/1121963. Accessed 12 August 2014

[103] Engelhardt M, Eber SW, Germing U, Heimpel H, Kern W, Schmugge M, Haas PS, Minkov M, Theilacker C (2013) Prävention von Infektionen und Thrombosen nach Splenektomie oder funktioneller Asplenie. DGHO Onkopedia. Available online at: http://www.dgho-onkopedia.de/de/onkopedia/leitlinien/praevention-von-infektionen-und-thrombosen-nach/pra-vention-von-infektionen-und-thrombosen-nach.pdf. Accessed 12 August 2014

[104] Robert Koch Institut (2013) Impfungen bei Asplenie (Entfernung der Milz oder Ausfall der Organfunktion). Robert Koch Institut. Available online at: http://www.rki.de/SharedDocs/FAQ/Impfen/AllgFr_ImpfGesundheitsschaden/FAQ01.html?nn=2391120. Accessed 12 August 2014

[105] Società scientifiche dell’Igiene pubblica, Federazione Italiana Medici Pediatri, and Federazione Italiana Medici di Medicina Generale (2012) Calendario Vaccinale per la Vita. Società scientifiche dell’Igiene pubblica. Available online at: http://www.societaitalianaigiene.org/site/new/images/docs/calendariovaccinale/2012/cvplv.pdf. Accessed 12 August 2014

[106] World Health Organization (WHO) (2005) Vaccine introduction guidelines. Adding a vaccine to a national immunization programme: decision and implementation. WHO. Available online at: http://whqlibdoc.who.int/hq/2005/WHO_IVB_05.18.pdf Accessed 12 August 2014

[107] Boccalini S, Bechini A, Levi M, Tiscione E, Gasparini R, Bonanni P (2013). Cost-effectiveness of new adult pneumococcal vaccination strategies in Italy. Hum Vaccin Immunother.

[108] Kuhlmann A, Theidel U, Pletz MW, von der Schulenburg JM (2012). Potential cost-effectiveness and benefit–cost ratios of adult pneumococcal vaccination in Germany. Health Econ Rev.

[109] Hak E, Grobbee DE, Sanders EA, Verheij TJ, Bolkenbaas M, Huijts SM, Gruber WC, Tansey S, McDonough A, Thoma B, Patterson S, van Alphen AJ, Bonten MJ (2008). Rationale and design of CAPITA: a RCT of 13-valent conjugated pneumococcal vaccine efficacy among older adults. Neth J Med.

[110] clinicaltrials.gov (2014) Study Evaluating the Efficacy of a 13-Valent Pneumococcal Conjugate Vaccine (13vPnC) in Adults (CAPITA). clinicaltrials.gov. Available online at: http://www.clinicaltrials.gov/ct2/show/NCT00744263?term=Prevenar+13&rank=92. Accessed 12 August 2014

[111] Myint TT, Madhava H, Balmer P, Christopoulou D, Attal S, Menegas D, Sprenger R, Bonnet E (2013). The impact of 7-valent pneumococcal conjugate vaccine on invasive pneumococcal disease: a literature review. Adv Ther.

[112] Myint TT, Madhava H, Balmer P, Christopoulou D, Attal S, Menegas D, Sprenger R, Bonnet E (2012) The impact of 7-valent pneumococcal conjugate vaccine (PCV7) on pneumonia and mortality: a literature review. Presented at the 8th International Symposium on Pneumococci and Pneumococcal Diseases, Iguaçu Falls, Brazil, March 11–15, 2012. Available online at: http://www2.kenes.com/ISPPD/Scientific/Documents/FinalAbstractbook.pdf. Accessed 12 August 2014

[113] Moore M, Link-Gelles R, Farley MM, Schaffner W, Thomas A, Reingold A, Harrison L, Lexau C, Zansky S, Petit S, Gershman K, Scherzinger K, MacInnes K, Beall B, Whitney C (2012) Impact of 13-valent pneumococcal conjugate vaccine on invasive pneumococcal disease, U.S., 2010–11. Presented at IDWeek 2012, San Diego, CA, October 17–21, 2012. Available online at: https://idsa.confex.com/idsa/2012/webprogram/Paper36569.html. Accessed 12 August 2014

[114] Picazo J, Ruiz-Contreras J, Casado-Flores J, Giangaspro E, García-de Miguel MJ, Hernández-Sampelayo T, Otheo E, Méndez C (2012) First impact data of 13-valent pneumococcal conjugate vaccine (PCV13) on invasive pneumococcal disease in children in Madrid, 2010–2011 (Heracles study). Presented at the 8th International Symposium on Pneumococci and Pneumococcal Diseases, Iguaçu Falls, Brazil, March 11–15, 2012. Available online at: http://www2.kenes.com/ISPPD/Scientific/Documents/FinalAbstractbook.pdf. Accessed 12 August 2014

[115] Georgakopoulou T, Menegas D, Tzanakaki G, Pipa E, Vernardaki A, Mavraganis P, Theodoridou M., Kremastinou J (2012) Epidemiology of bacterial meningitis in Greece, in the era of conjugate vaccines: a 7 years review 2005–2011. Presented at the 52nd ICAAC, San Francisco, CA, USA, September 9–12 2012. Available online at: http://www.abstractsonline.com/Plan/ViewAbstract.aspx?sKey=d201a978-86b7-4126-b4eb-dce82709451f&cKey=fd9b920f-2dad-4c23-9dba-3f0f20b33da8&mKey=%7b6B114A1D-85A4-4054-A83B-04D8B9B8749F%7d. Accessed 12 August 2014

[116] Pardo-Sanchez F, García-Magan C, Martinon-Torres N, Guinda Gimenez M, Méndez-Lage S, Seoane-Pilado MT, Pertega-Diaz S, Martinon-Torres F (2012) Early impact of a universal immunization program with the 13-valent conjugate pneumococcal vaccine in Santiago de Compostela area (Spain). Abstract 588. Presented at the 30th Annual Meeting of the European Society for Paediatric Infectious Diseases, Thessaloniki, Greece, May 8–12 2012. Available online at: http://espid.kenes.com/Documents/ESPID2012%20ABSTRACTS.pdf. Accessed 12 August 2014

[117] Pichichero M, Casey JR, Center K, Chen L, Mathur P, Durham K, Gurtman A, Scott DA (2012) Efficacy of PCV13 in prevention of AOM and NP colonization in children: first year of data from the US. Presented at the 8th International Symposium on Pneumococci and Pneumococcal Diseases, Iguaçu Falls, Brazil, March 11–15, 2012. Available online at: http://www2.kenes.com/ISPPD/Scientific/Documents/FinalAbstractbook.pdf. Accessed 12 August 2014

